# Selection of Pediatric Mental Health Quality Measures for Health System Improvement in British Columbia Based on a Modified Delphi Approach

**DOI:** 10.3389/fped.2022.866391

**Published:** 2022-07-06

**Authors:** Sina Waibel, Wan Ling Wu, Michael Smith, L. Kit Johnson, Rita D. Janke

**Affiliations:** ^1^Child Health BC, Provincial Health Services Authority, Vancouver, BC, Canada; ^2^Department of Pediatrics, Faculty of Medicine, University of British Columbia, Vancouver, BC, Canada; ^3^Fraser Health, New Westminster, BC, Canada

**Keywords:** health care quality indicators, quality improvement, substance-related disorders, Delphi techniques, pediatrics, mental health services

## Abstract

**Background:**

The COVID-19 pandemic has highlighted the importance of mental wellbeing. The identification and implementation of quality measures can improve health outcomes and patient experience. The objective was to identify and define a core set of valid and relevant pediatric mental health quality measures that will support health system evaluation and quality improvement in British Columbia, Canada.

**Methods:**

The study consisted of four phases. First, a comprehensive database search identified valid pediatric quality measures focused on mental health and substance use (MH/SU). Second, the identified quality measures were mapped to focus areas, which were then prioritized by two stakeholder groups consisting of 26 members. Third, up to two representative measures for each prioritized focus area were pre-selected by an expert panel (*n* = 9). And fourth, a three-step modified Delphi approach was employed to (1) assess each quality measure on a 7-point Likert scale against three relevance criteria (representative of a quality problem, value to intended audience and actionable), (2) discuss the results, and (3) select and rank the most relevant measures. Forty-eight stakeholders were invited to participate; of those 24 completed the round 1 survey, 21 participated in the round 2 discussion and 18 voted in the round 3 selection and ranking survey. For round 1, consensus was determined when at least 70% of the response rates were within the range of five to seven. For round 3, Kendall's coefficient of concordance W was used as an estimator of inter-rater reliability.

**Results:**

One-hundred pediatric mental health quality measures were identified in the database search. Of those, 37 were mapped to ten focus areas. Pre-selection resulted in 19 representative measures moving forward to the Delphi study. Eleven measures met the consensus thresholds and were brought forward to the round 2 discussion. Round 3 ranking showed moderate to strong raters' agreement (Kendall's W = 0.595; *p* < 0.01) and resulted in the following five highest-ranked measures: level of satisfaction after discharge from inpatient admission due to MH/SU, number of patients experiencing seclusion or restraint, length of time from eating disorder referral to assessment, number of ED visits due to MH/SU, and number of readmissions to ED.

**Conclusion:**

The selected core set of valid and relevant pediatric quality measures will support sustainable system change in British Columbia. The five top-ranked measures will be refined and tested for data collection feasibility before being implemented in the province.

## Introduction

Mental wellbeing and social and emotional development are fundamental to human development and essential for all children to flourish (1). Investing in children and youth can improve health and wellbeing both in midlife and in later years (2). However, many Canadian children experience mental health problems that are serious enough to interfere with their development and impair their functioning (3). These problems include emotional difficulties, such as depression and anxiety, and behavioral difficulties, such as aggression, inattentiveness, and hyperactivity (3). Limited access to care and the mal-distribution of providers as well as the lack of coherent policies, impede the adequate delivery of mental health care to children and youth (4).

The mental health of children and youth has gained increased attention since the WHO declared in March 2020 the coronavirus disease 2019 (COVID-19) outbreak to be a pandemic (5). Public health measures aimed at slowing down the viral spread, such as school closure or change and disrupting of social networks and access to community activities, have contributed to unintended societal consequences such as poorer mental health (6). Children are not the face of this pandemic, but they are considered its “biggest victims” as the COVID-19 crisis has a profound effect on their wellbeing (7). The withdrawal from social life and daily activities such as attending school, combined with fear, anxiety and the feeling of unpredictability, increase the risks for this group to develop psychiatric disorders in the future, even for those who do not have such histories (8).

Twenty percent of the total population in the province of British Columbia, Canada, are children and youth from 0 to 19 years. The highest number of children live in the Lower Mainland (the region surrounding and including Vancouver), but the highest ratio of children to adults is found in the rural and northern parts of British Columbia, where 24% of the total population are children (9). An estimated 18,600 pediatric emergency department (ED) visits each year are related to mental health or substance use complaints. This represents 4% of the total pediatric ED visits. Of this group, approximately one quarter are admitted (10). Depressive episodes, reactions to severe stress and adjustment disorders, and eating disorders are responsible for nearly half of the pediatric inpatient admissions in British Columbia (11).

Community reports also show a background increase in mental health problems. Results of a survey of youth aged 12–19 in British Columbia showed an increase in self-reported mental health conditions by both male (5% in 2013 vs. 8% in 2018) and female adolescents (15 vs. 23%). When asked about specific mental health conditions, the participants reported suffering from anxiety disorder or panic attacks (19%), depression (15%), or attention-deficit/hyperactivity disorder (7%) (12). It is expected that increased childhood mental health problems have emerged since the onset of the Covid-19 pandemic and are predicted to continue (13, 14).

In British Columbia, five regional or geographic and two provincial health authorities (Provincial Health Services Authority, and First Nations Health Authority) administer hospital or community-based services or both; either by delivering the services directly or by contracting with other health care organizations and providers (15). The responsibilities for the delivery of mental health services for children and youth, as in other provinces in Canada (16), are shared across different governmental ministries: the Ministry of Children and Family Development through community-based and residential services, the Ministry of Health through hospitals and ambulatory services, and the Ministry of Education through public education and student services. Furthermore, the Ministry of Mental Health and Addictions has responsibility for policy development, program evaluation and research in relation to mental health and addiction services across the lifespan. Since mental health and substance use care had not been a priority of any provincial government until recently, services are fragmented and lack the consistency of oversight and delivery (17).

Child Health BC is a provincial health improvement network under the Provincial Health Services Authority that brings together child and youth leads from across the province to promote shared learning, innovation and quality improvement, and to deliver a more integrated system of care using the Tiers of Service framework (18). This framework aims to support provincial collaboration and provide a consistent and standardized approach to service planning and delivery across the province. Services, such as children's mental health (Supplementary Figure 1; Supplementary Table 1), emergency care, critical care, or surgery, are categorized as Tiers 1 through 6. Tier 1 offers a wide breadth of service that is accessible in most communities, targeting health promotion and common, low complexity health needs across the life span. In comparison, Tier 6 offers in-depth, sub-specialized pediatric-focused services targeting low incidence, high complexity and acuity health needs, which often require the coordination with other on-site subspecialty teams (18).

In recent provincial cross-sectional studies that aimed to analyze the availability of pediatric services related to emergency care, critical care and mental health, and that applied the Tiers of Service framework (18), the need to establish structures and processes to track provincial child and youth specific health quality measures was identified. Although health provider organizations in British Columbia submit a standard set of data elements to the Canadian Institute of Health Information (CIHI), which are then summarized and made publically available, the time lag between data submission and access hinders the usefulness of the data source to support timely quality improvement, continuous system learning and sustainable system change (19).

It has been recognized that “quality measurement is a critical tool for improving healthcare quality and patient safety” (20). A pediatric quality measure provides a reference point to which data on child health care service provision can be assessed and quantified against clear evidence-based criteria in terms of its quality domains [safety, effectiveness, patient-centeredness, timeliness, equity, and efficiency (21, 22)]. A quality measure includes the methods required to determine the performance of a quality indicator, linking evidence-based outcomes with health system structures or processes (22). Quality measures can be specific to conditions, such as treatment of asthma, or cut across conditions, such as coordination of care or hospital readmission (20). Measures need to be carefully selected or developed based on the pertinent quality issue, knowledge of the stakeholders and the purpose of measurement (23, 24).

Much of the resources allocated to the development of quality measures have been targeted toward the adult population and only small investments in child health quality measure development have been made (20). By identifying and implementing a core set of measures and developing supportive quality and reporting structures, there is potential to improve the health outcomes and experience of the child and youth population. The Child Health BC Provincial Quality Committee, which is supported and co-chaired by Child Health BC, was tasked with guiding the development of a core set of quality measures for use within British Columbia. The objective was to identify and define a core set of valid and relevant pediatric mental health quality measures that will support health system evaluation and quality improvement in the province.

## Materials and Methods

**Figure 1:**
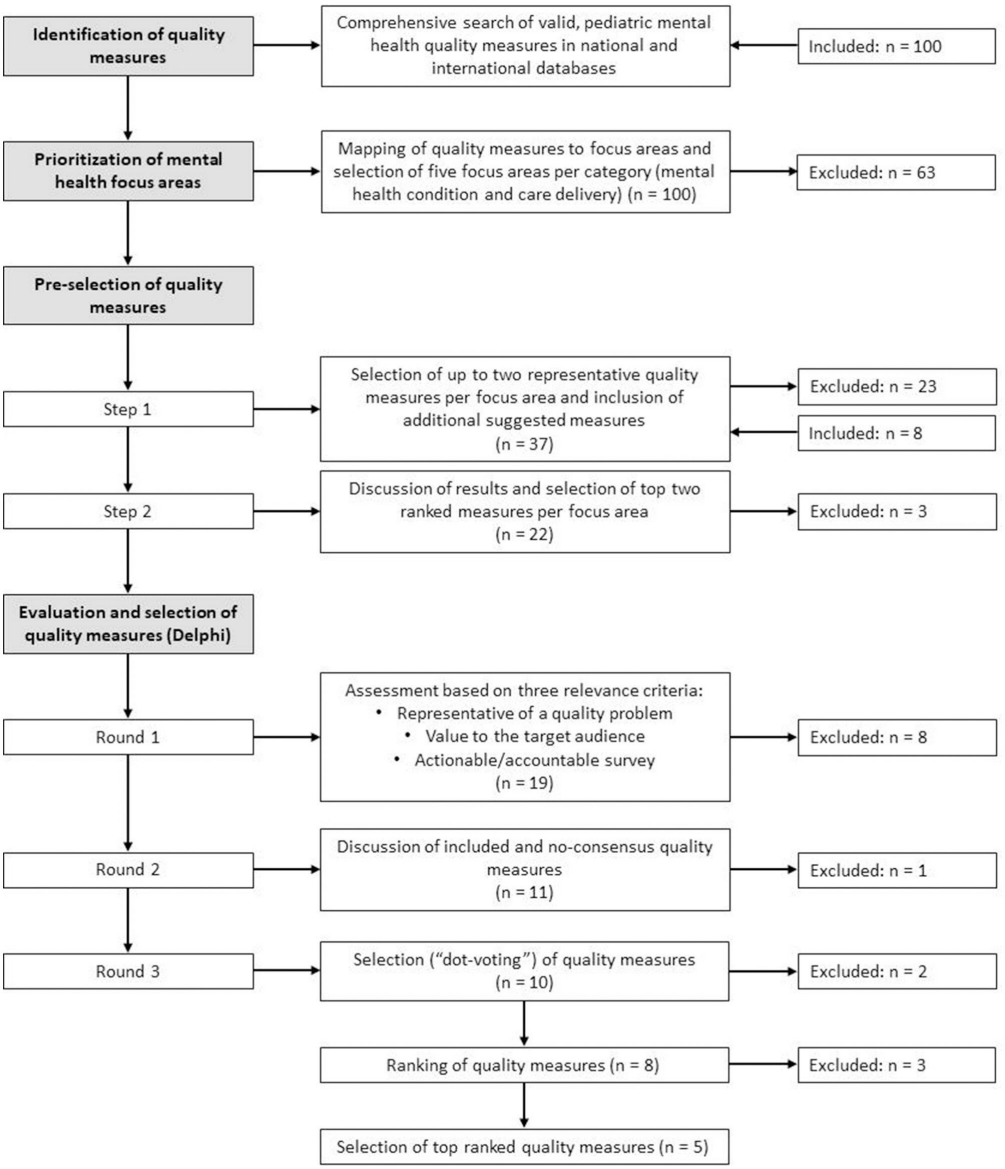
Flow chart of the different study phases.

The study consisted of four phases: identification of quality measures, prioritization of mental health focus areas, pre-selection of quality measures, and evaluation and selection of quality measures using Delphi techniques. The study was conducted between October 2020 and May 2021. The different phases are outlined in Figure 1.

### Participants

The first phase, the identification of quality measures, was conducted by the research team that consisted of a health policy researcher, a quality improvement specialist, a health data analyst and a pediatric mental health clinician. For the Delphi study, members of two pre-existing provincial stakeholder groups (Child Health Provincial Quality Committee, and Mental Health Tiers of Service Advisory Group) were invited to participate. The provincial stakeholder groups had representation from child health leaders, health professionals and researchers across the province within the areas of quality improvement, mental health, emergency care and pediatric care. In addition, representation of key stakeholders from different health authorities was sought during the selection process, as well as youth and family with lived experience, to ensure that a heterogeneous group was represented (25). A total number of 48 participants were invited to participate in the Delphi study. For the two study phases preceding the Delphi study, smaller expert panels consisting of a subset of the broader stakeholder group were asked to participate (*n* = 26 for the prioritization of mental health focus areas and *n* = 9 for the pre-selection of quality measures). Diverse roles were represented in the expert panel that consisted of nine members: psychiatrist, psychologist, social worker, pediatrician, pediatric emergency physician, clinical nurse specialist, child and youth mental health researcher, mental health operational leader and mental health quality improvement director. A small number of participants from private practice received a clinical sessional reimbursement for their involvement.

### Identification of Quality Measures

A comprehensive database search was conducted to identify pediatric mental health quality measures. The search was based upon the search strategy identified by Woolfenden et al. (22), who conducted a comprehensive review on pediatric quality measures (i.e., not specific to mental health) and identified organizations and initiatives for quality measure development testing and endorsement in the USA, Australia, United Kingdom and the European Union. We then searched for similar organizations and initiatives from Canada. The final list included for example the National Quality Forum (NQF), the Agency for Health Care Research and Quality (AHRQ) or Medicaid, the National Institute for Health and Care Excellence (NICE), and the Canadian Institute for Healthcare Improvement (see Supplementary Table 2 for the complete list). To identify relevant quality measures in the databases, a comprehensive search strategy consisting of a combination of descriptors and keywords related to the research area was utilized. Used search words included but were not limited to mental health, behavioral health, substance use, ADHD, depression, anxiety, self-harm, suicide, psychotic disorders, and eating disorders.

A file of relevant indicators was built that met the following inclusion criteria: (i) focus on mental health and substance use, (ii) have been validated [i.e., have undergone significant testing to ensure that the indicator measures what is meant to measure and is free from random and systemic error (22)] or have been used within large quality measurement programs, and (iii) assess services provided through hospital-based care (ED, inpatient, and outpatient). Measures assessing primary or community care as well as sexual health were excluded from the study. A core set of mental health primary care measures will be identified at a later point in time; hospital care was chosen as the first area of focus since we expected that the advisory group established for a recently conducted provincial cross-sectional study on hospital-based mental health care would support eventual implementation of the selected quality measures. Since a significant number of pediatric mental health quality measures were identified in the database search, we discarded the need for a further comprehensive medical and gray literature review.

### Prioritization of Mental Health Focus Areas

Given the broad scope of child and youth mental health conditions, service delivery structures and processes, there was a need to identify key areas of focus that could guide the prioritization of mental health quality measures. The research team grouped and mapped the quality measures retrieved from the database review into focus areas associated with two discreet domains: specific mental health conditions (including reason for seeking care) and care delivery processes. Participants of the two broad stakeholder groups were asked to identify additional focus areas that might have been missed and then rank them, from a quality of care perspective, depending on their perceived relevance for the province. The ranking should result in the selection of four focus areas in each of the domains. Mentimeter, an online interactive real-time voting tool, was used to facilitate the identification and ranking process. Three rounds of ranking with discussion in between each round were conducted.

### Pre-selection of Quality Measures

The quality measures identified in the database search and linked to the focus areas underwent a two-step pre-selection process to streamline them to a manageable number for the next study phase (the broad stakeholder Delphi rounds) and ensure that the most relevant measures were put forward. In the first step, a small expert panel was asked to choose up to two representative measures for each of the mental health focus area, using an online survey tool called REDCap (Research Electronic Data Capture); which is a secure web application for building and managing online surveys and databases (26). The survey also allowed the experts to suggest modifications to the presented measures and include additional valid measures. These were then researched to determine whether similar measures were already being used in other jurisdiction and had perhaps been missed by the database review. Focus areas with two or fewer measures were automatically brought forward. In the second step, a virtual meeting was organized to discuss the results and rank the measures using Mentimeter. The top two ranked measures per focus area were selected for the next phase.

### Evaluation and Selection of Quality Measures Using Delphi Techniques

#### Study Design

A three-step modified Delphi approach was used to evaluate and select a final set of provincial mental health quality measures. Delphi techniques are systematic approaches that support decision-making and are commonly used to identify quality indicators or measures (27–30). In mental health research, Delphi techniques often answer questions that may not be possible or feasible with alternative methodologies (25). Delphi techniques allow for input from a variety of individuals, mitigating some of the risks of bias that can be associated with group decision-making and the influence of dominant voices (28, 31). A series of questionnaire surveys called rounds are held until a predetermined consensus threshold and stability are obtained. Participants in the Delphi process are typically experts in the field in consideration. It has also been suggested to include lay people such as patients with lived experience (28). We applied a modified approach by giving the panelists the chance to discuss their answers and the results between the rating rounds (32).

#### Data Collection

Members of the two provincial stakeholder groups were invited by e-mail to participate in the modified Delphi selection process. They were provided with a guide that included information about the study purpose, overall Delphi process, the three relevance criteria and detailed technical notes on each of the pre-selected quality measures.

For round 1, an anonymous survey instrument was developed in REDCap© (26). Participants were asked to assess each of the quality measures against three relevance criteria (29, 33):

- *Representative of a quality problem in British Columbia*, defined as the extent of the quality problem addressed by the measure being substantial and including prevalence, risk and variation;
- *Value to the target audience*, defined as the quality problem being important to the intended audience (patients and/or providers); and
- *Actionable/accountable*, defined as programs/providers being clearly accountable for the quality problem assessed by the measure and having the ability and resources to improve their performance on the measure with the implementation of quality improvement efforts.

The assessment was based on a 7-point Likert scale: 1 = strongly disagree, 4 = neither disagree nor agree, and 7 = strongly agree that the indicator represents a quality problem in British Columbia, is of value to the target audience, and is actionable/accountable. Participants were informed that the selected measures would be adjusted to the British Columbian context and refined during the testing phase. For each measure, an open text box was available for the participants to comment on their rating if desired. Furthermore, a question about the perceived feasibility of data collection with a binary response option was added for each measure. An open-ended question was branched to the non-feasible response providing the participant with the opportunity to describe how data could be collected. Written consent was given by filling in the survey. Participants were encouraged to download their results at the end of the survey. The aggregated results and a summary of the comments were sent to all invitees, along with a matrix demonstrating how the seven BC quality dimensions (accessibility, appropriateness, effectiveness, efficiency, equity, respect and safety) (34) related to the measures. This step enabled participants to see where their responses stood in relation to that of the group (35) and supported preparation for the subsequent rounds.

For round 2, a 1.5 h virtual conference was organized to present the results from the first round and provide participants with an opportunity to voice their views on the selected measures, providing rationales why this measure should or should not be moved forward to the next study phase. The discussion prompted participants to reassess, alter or develop opinions regarding the proposed measures (32). No attempt was made to force the panel to consensus (32); rather the purpose of the discussion was to support the individual's decision-making on the relevance of the measure, which was assessed in the third and final Delphi round. A virtual session was utilized primarily to comply with Covid-19 pandemic regulations, which prohibited in-person meetings of large groups, but also to facilitate the inclusion of opinions from experts living in diverse geographic locations.

In round 3, which took place at the end of the virtual conference, participants were asked to select the five most relevant measures for use in British Columbia, taking into consideration current provincial mental health priorities (17), areas of care addressed (emergency care, inpatient, outpatient) as well as assigned BC quality dimensions (34). The intent was to only select five measures given the significant burden of provincial data collection and reporting of each measure once being implemented. Each selected measure was equally weighted [similar to “dot-voting” as employed in other studies (36–38)] using Mentimeter. This process allowed each participant to cast their votes anonymously and non-verbally, giving equal weight to each participant in the final determination of measures (38). Results of the selection were presented back to the panel during the session using the voting tool. They were then asked to provide feedback on the results and to discuss any concerns regarding the five measures with the highest votes. A final ranking round of the top eight measures was conducted to confirm the prioritization of the final five measures, allowing for differentiating the degree of importance between the items (39).

#### Data Analysis

Survey results of the first Delphi round were analyzed by computing measures of central tendency, quartiles, and frequency distributions for each quality measure by relevance criterion. Agreement percentages (consensus) were calculated by assessing if at least 70% of the response rates for each measure and relevance criterion were within the range of five to seven. A composite median score of all three relevance criteria was calculated for each measure to support prioritization. This score took into consideration different weights that were assigned to the relevance criteria based on a point allocation method (40, 41) conducted by the quality committee members (representative of a quality problem in British Columbia: 44.6%; value to the target audience: 28.9%; actionable/accountable: 26.7%). The following cut-off scores were determined for the first Delphi round (42, 43):

- Inclusion: median score of ≥5 on all three relevance criteria with ≥70% consensus.
- No consensus: weighted median composite score ≥6 and do not meet inclusion threshold.
- Exclusion: do not meet either of the above thresholds.

It was decided a priori that measures in the inclusion and no consensus categories would be retained and advance to the next round, while the remaining measures would be excluded. Feasibility results were not counted in the cut-off thresholds since it was decided that measures will be selected independently of the efforts required to collect them; however, feasibility results will be used as a proxy to understand data collection and testing workability in the next study phase. Open-ended sections were analyzed using thematic content analysis.

The round 2 discussion was recorded and participants' comments on the measures were summarized.

For round 3, the number of votes that each measure received and percentages were calculated. For ranking calculation, we examined how often an item was ranked at each position (frequencies of ranks) and then weighted exponentially the number of times an item was ranked at a certain position (39). Kendall's coefficient of concordance W was used as an estimator of inter-rater reliability (agreement on ranking across raters) (44). Rankings with missing data were omitted (*n* = 5). Statistical analyses were carried out in RStudio.

## Results

### Identification of Quality Measures

One-hundred valid pediatric mental health quality measures were identified from the national and international database and dataset search. The majority of quality measures and standards came from the National Institute for Health and Care Excellence (NICE) (*n* = 33), the Ontario Mental Health of Children and Youth 2017 Status Report (*n* = 17), the Agency for Health Care Research and Quality (AHRQ) (*n* = 16) and the National Quality Forum (NQF) (*n* = 13). The most frequently identified quality measures were eating disorders (*n* = 13) and mood disorders (depression, bipolar) (*n* = 12), since there are existing quality standards from organizations such as NICE and Health Quality Ontario. Only a few measures were identified in areas such as psychotic and schizophrenic disorders or disruptive behavior. Fourteen measures focused on access, including subspecialty access to mental health services and eight on transition and discharge (Supplementary Table 2).

### Prioritization of Mental Health Focus Areas

The 100 identified measures were assigned by the research team to nine focus areas related to the mental health conditions domain (including reasons for seeking care) and nine focus areas related to the care delivery domain. Stakeholders added one additional condition to the mental health condition domain: neurodiverse conditions including autism. Differences in the ranking between the two groups resulted in the decision to include five focus areas in each domain. The following focus areas were selected in the mental health conditions: domain (by highest number of ranking) (1) self-harm, (2) substance use including substance-related disorders, (3) eating disorders, (4) behavioral disorders including conduct disorders, oppositional defiant disorders, and externalizing behavior disorders, and (5) neurodiverse conditions; and in the care delivery domain: (1) access/subspecialty access, (2) provider education including training, clinical supervision and support, (3) transitions including discharge and follow up care, (4) restraint and seclusion, and (5) psychosocial care and family functioning. Thirty-seven measures were associated with the selected focus areas and the remaining 63 were excluded from the study (see Supplementary Table 2).

### Pre-selection of Quality Measures

Out of the 37 measures, 14 were selected by the expert group through the online survey and moved forward to the discussion and ranking round together with 8 additional suggested measures. One of the additional measures was similar to a measure identified previously in the database search; we therefore added the one from the search back in. The discussion and ranking process resulted in the selection of 19 measures; out of these 13 had been identified in the original database search and 6 came from the 8 additional measures suggested by the experts (see Supplementary Table 2).

### Evaluation and Selection of Quality Measures Using Delphi Techniques

#### Participants

The round 1 questionnaire was sent to 48 stakeholders, with a response rate of 50% who completed part of (*n* = 4) or the whole survey (*n* = 20). Of those, 21 stakeholders participated in the round 2 discussion and 18 voted in the round 3 selection and ranking survey. We assumed that only those individuals who completed the round 1 survey also participated in the proceeding rounds. Most of the participants were female (67 and 50% in round 1 and 3, respectively) and were between 40 and 49 years old (46 and 44%). Over 40% of participants had >20 years of clinical experience. Most worked as administrators or clinicians (67 and 78%), with half of all participants or less focusing on mental health care (50 and 35%). Patient and family partners also actively participated in the Delphi study (8 and 17%) (Table 1).

**Table 1:** Characteristics of participants of Delphi rounds 1 and 3.

	**Round 1: *n* (%)**	**Round 3: *n* (%)**
**Gender**		
Male	4 (16.7)	7 (38.9)
Female	16 (66.7)	9 (50.0)
Prefer not to say	4 (16.7)	2 (11.1)
**Age**		
Under 29 years	1 (4.2)	2 (11.1)
30–39 years	2 (8.3)	1 (5.6)
40–49 years	11 (45.8)	8 (44.4)
50–69 years	7 (29.2)	6 (33.3)
Over 70 years	0 (0.0)	0 (0.0)
Prefer not to say	3 (12.5)	1 (5.6)
**Years of experience or work experience with health care services**		
<5 years	1 (4.2)	0 (0.0)
5–9 years	3 (12.5)	4 (22.2)
10–19 years	9 (37.5)	6 (33.3)
More than 20 years	11 (45.8)	8 (44.4)
**Role/main responsibility in current position**		
Administrator	10 (41.7)	7 (38.9)
Clinician	6 (25.0)	7 (38.9)
Researcher	1 (4.2)	0 (0.0)
Parent, caregiver, patient	2 (8.3)	3 (16.7)
Others	5 (20.8)	1 (5.6)
Role/experience predominately in mental health*		
Yes	12 (50.0)	6 (35.3)
No	12 (50.0)	11 (64.7)

*5.6% missing in round 3. No demographic data was collected for the round 2 discussion.

#### Round 1: Evaluation of Quality Measures

Three measures achieved a median score of ≥5 on all three relevance criteria with ≥70% consensus and thus were for inclusion (N°6, 16 and 18). They related to the focus areas eating disorders, substance use and follow up. Eight quality measures had a weighted median composite score ≥6 but did not meet the inclusion threshold and were therefore assigned to the no consensus category (N°4, 5, 10, 11, 13-15 and 17). The remaining 8 measures did not meet any thresholds and were thus excluded (Table 2). The two measures that reached the highest composite score of 6.7 referred to substance use and discharge (N°15 and 17). The median score for the two relevance criteria *representative of a quality problem in British Columbia* and *value to the target audience* was 6.0 (range: 5.0–7.0), while the median score for *actionable/accountable* was slightly lower (5.5, range: 5.0–7.0). Comments in the open text boxes were dominated by remarks about the feasibility of data collection: “*This may be difficult to collect [documentation in discharge plan about communication between acute and outpatient providers]. Chart audits require resource supports to put in place consistently. Nursing leads and educators do not have capacity to add audits to their workload.”* (Administrator, female about measure N°14) or the importance of tracking the measure across the province: “*This is a very important indicator [number of patients experiencing seclusion or restraint] due to the multiple safety risks (emotional and physical) that go along with restraint and seclusion, for patients, staff and families.”* (Administrator, female about measure N°13).

**Table 2:** Results of Delphi rounds 1 and 3.

**Quality measure**		**Focus area**	**BC quality dimension**	**Round 1**							**Round 3**		
				**Quality problem in British Columbia**		**Value to the target audience**		**Actionable/accountable**		**Composite score**	**Decision**	**Selection**	**Ranking**
				**Consensus (%)**	**Median score**	**Consensus (%)**	**Median score**	**Consensus (%)**	**Median score**	**Median score**		**Votes *n* (%)**	**Order**
1	Number of emergency department (ED) visits of children/young people whose presenting complaint is behavior*	Access/ sub-specialty access	Effectiveness, appropriateness	78%	6	78%	6	43%	4	5.5	Excluded	-	-
2	Number of children/young people brought to the ED by police, under Section 28 of the Mental Health Act (45), where the presenting complaint is behavior due to a mental health disorder*	Access/ sub-specialty access	Safety, respect	71%	6	74%	6	48%	4	5.5	Excluded	-	-
3	Number of children/young people admitted who have pre-existing community supports*	Access/ sub-specialty access	Access	75%	6	79%	6	50%	4.5	5.6	Excluded	-	-
4	Rate of ED visits related to mental health and substance use for aged 0 to 18.9 years overall, by years, by gender and Tiers of Service [adjusted from (46)]**	Access/ sub-specialty access	Access, appropriate ness	85%	6.5	84%	6	65%	6	6.2	No consensus	14 (16%)	4
5	Rate of admission related to mental health and substance use for aged 0-18.9 years overall, by years, by gender and Tiers of Service [adjusted from (46)]	Access/ sub-specialty access	Access, equity	90%	6.5	84%	6	65%	5.5	6.1	No consensus	5 (6%)	6
6	Length of time from referral to assessment and start of treatment at an eating disorder service for children/young people with suspected eating disorders (47)	Eating disorders	Access	91%	6	95%	6	86%	6	6.0	Inclusion	16 (18%)	3
7	Number of admissions for eating disorders per 10,000 population aged 0 to 18.9 years, overall, by gender and Tiers of Service [adjusted from (46)]	Eating disorders	Effectiveness, safety	86%	6	76%	6	64%	5	5.7	Excluded	-	-
8	Proportion of children/young people with neurodiverse conditions admitted with behavior as the primary reason for admission who are assessed for possible triggers, including physical health conditions, mental health problems and environmental factors [adjusted from (47)]	Neurodiverse conditions	Safety, effectiveness, appropriateness	68%	5	78%	6	67%	5.5	5.4	Excluded	-	-
9	Proportion of children/young people with neurodiverse conditions and challenging behavior who are prescribed antipsychotic medication for the treatment of their behavior in whom psychosocial interventions are insufficient or cannot be delivered because of the severity of the behavior [adjusted from (47)]	Neurodiverse conditions	Appropriateness	72%	5	81%	5	56%	5	5.0	Excluded	-	-
10	Percentage of staff that have completed the Mental Health Act online module or face-to-face session [adjusted from (45)]	Provider education	Effectiveness, safety, respect	70%	6	68%	6	75%	7	6.3	No consensus	2 (2%)	-
11	Percentage of staff that have completed training in least restraints guidelines as per health authority (initial and annual training)*	Provider education	Safety, effectiveness, respect	60%	6	68%	6	70%	6	6.0	No consensus	9 (10%)	8
12	Proportion of children/young people whose parents/caregivers were assessed for psychosocial wellbeing [adjusted from (48)]	Psychosocial/ family functioning	Respect, safety	65%	5	68%	6	47%	4	5.0	Excluded	-	-
13	Total number of children/young people who experienced at least one event of seclusion or restraint (physical and chemical) during their stay: (a) number of children/young people who experienced at least one seclusion, restraint or both; (b) number of seclusions, restraint or both events per patient stay*	Restraint/ seclusion	Safety, effectiveness, appropriateness	75%	6.5	74%	7	60%	5	6.3	No consensus	15 (17%)	2
14	Number of children/young people discharged with self-harm who have documentation in the discharge plan that showed communication and planning between acute and outpatient providers for discharge follow-up [adjusted from (48)]	Self-harm	Safety, appropriateness	86%	6	85%	6	67%	6	6.0	No consensus	5 (6%)	7
15	Percentage of ED visits with presenting complaints of substance use who had a follow-up visit for substance use within 7 and/or 30 days of the ED visit. Two rates are reported: 7 and 30 days [adjusted from (49)]	Substance use	Access, safety, effectiveness	85%	7	95%	7	68%	6	6.7	No consensus	4 (4%)	-
16	Proportion of children/young people who visited the ED for a substance-related disorder by age, health authorities, Tiers of Service, rural/urban residence [adjusted from (50)]**	Substance use	Access, appropriateness	85%	6.5	89%	7	70%	5.5	6.4	Inclusion	14 (16%)	4
17	Level of satisfaction with support following discharge from an inpatient admission for mental health/substance misuse [adjusted from (47)]	Transition/ discharge/ follow up	Respect, appropriateness	95%	7	90%	7	68%	6	6.7	No consensus	15 (17%)	1
18	Number of readmissions to the ED within 30 days with a presenting mental health or substance use complaint [adjusted from (46)] *	Transition/ discharge/ follow up	Safety, access	80%	6.5	79%	6	70%	6	6.2	Inclusion	5 (6%)	5
19	Unplanned readmissions to inpatient mental health services within 30 days of a mental health inpatient discharge (47)	Transition/ discharge/ follow up	Effectiveness, safety	86%	6	90%	6	68%	5.5	5.9	Excluded	-	-

*Added by the expert panel. ^**^Quality measures were combined. Consensus (%) of response rates with median score of ≥ 5. Composite score is the weighted median score of all three relevance criteria. Sources: BC Quality Dimension (34); Tiers of Service (18).

#### Round 2: Discussion

Participants commented on the usefulness and relevance of each measure, for example, by questioning if measures assessing provider education and training would change behaviors and improve mental health care delivery. They also provided rationales why a specific measure should be moved forward to the next study phase; for example, international benchmarks are available for measures related to eating disorders, which would facilitate the discussion about setting targets. Further, the group acknowledged the importance of having patient and family experience represented in the final set of measures to ensure that their voice is heard and drives improvement within the system, as this would be the case for measure N°17. Finally, participants highlighted the need to provide clear definitions (for instance, about self-harm), provided suggestions to improve or expand some of the measures (which will be considered in the testing phase), and proposed to merge measures N°4 and 16 since both analyze ED visits for mental health or substance use concerns and data disaggregation can be used to specify the reason for the visit.

#### Round 3: Selection and Ranking of Quality Measures

The measures that received the highest number of votes related to eating disorder service wait times (N°6, 18%), number of patients experiencing restraint or seclusion (N°13, 17%), satisfaction with discharge (N°17, 17%), and ED utilization (N°16, 16%) (Table 2). The two measures with the lowest votes related to staff education about the Mental Health Act (45) (N°10, 2%) and ED follow-up visit for substance use (N°15, 4%) and were removed, resulting in 8 measures to move forward to the discussion round and final ranking process. The five highest-ranked measures referred to two focus areas in the mental health conditions domain (eating disorders and substance use) and three in the care delivery domain (access/subspecialty access, transition/discharge/follow up, restraint/seclusion). Two measures related to transition, while one measure, which was combined in round 2, referred to both substance use and access. The following five measures were selected: level of satisfaction after discharge for mental health or substance use (N°17, 105 points), number of patients experiencing seclusion or restraint (N° 13, 101 points), length of time from eating disorder referral to assessment (N°6, 100 points), proportion of patients who visited the ED for mental health or substance use (N°4 and 16, 95 points) and number of readmissions to ED due to mental health or substance use (N°18, 68 points) (Table 3). The raters' agreement on the ranking was moderate to strong using Smith's (44) interpretation (Kendall's W = 0.595; *p* < 0.01).

**Table 3:** Frequencies of ranks and point allocation of quality measures.

**Quality measure**	**Focus area**	**1^**st**^**	**2^**nd**^**	**3^**rd**^**	**4^**th**^**	**5^**th**^**	**6^**th**^**	**7^**th**^**	**8^**th**^**	**Points**
N°17: Level of satisfaction after discharge from inpatient admission for mental health or substance use	Transition/discharge/follow up	4	5	2	3	2	1	0	0	105
N°13: Number of patients experiencing seclusion or restraint	Restraint/seclusion	4	5	2	2	3	0	0	0	101
N°6: Length of time from eating disorder referral to assessment	Eating disorders	2	2	7	5	0	1	0	0	100
N°4 and 16: Proportion of patients who visited the ED for mental health or substance use	Access/sub-specialty access and substance use	5	2	5	1	1	0	1	0	95
N°18: Number of readmissions to ED due to mental health or substance use	Transition/discharge/follow up	0	1	1	5	5	2	2	0	68
N°5: Rate of admission for mental health and substance use	Access/sub-specialty access	2	1	0	0	4	5	1	2	58
N°14: Number of patients with documented discharge plan for self-harm follow up	Self-harm	0	1	0	1	1	2	5	3	35
N°11: Percentage of staff with training in least restraints guidelines	Provider education	0	0	0	0	0	3	3	7	22

*Please see Table 2 for the complete name of the quality measure*.

## Discussion

Mental health and wellness are fundamental to healthy child development (51). Early onset of mental illness and delay in or lack of access to adequate interventions frequently result in a downward spiral of disadvantage and suffering for young people and their families (52). Measurement is a key component of advancing health care quality, allowing for meaningful comparisons across institutions and providers to drive improvement (53). Although the development of pediatric-specific quality measures has intensified, it has not kept pace with the number and breadth of quality measures applicable to adults (20). The use of formal methods such as Delphi has been suggested in research dealing with highly complex systems and slowly changing consensus, as is the case with mental health (25). We employed a modified Delphi approach to define a core set of child and youth mental health quality measures that will be implemented across the province of British Columbia.

The final selected measures were linked to five focus areas. There was at least one measure chosen in each of the highest-ranked focus areas, except for self-harm and provider education. Several contextual factors may have impacted the selection of the specific measures and its linked focus areas. Firstly, there is currently no provincial data sharing plan to support timely access to pediatric health data across the province. This was highlighted through the experience with the COVID-19 pandemic, where understanding provincial patterns of ED and inpatient utilization was deemed important. The participant discussion focused on the necessity to establish processes to support timely provincial availability of foundational access measures, such as the number of ED visits or admissions by condition, rather than the use of condition-specific measures at this time. Secondly, a perceived increase in the number and severity of children and youth being seen and admitted with eating disorders heightened the need to include a quality measure that demonstrated access and flow of mental health services for this sub-population. And thirdly, patient and family participants emphasized the importance to include a measure that demonstrated the use of seclusion and restraint within the province. This was most likely due to two reasons. First, a recent provincial report on the rights of children and youth under the BC Mental Health Act (45) highlighted the negative impact of restraint use in the province. And second, concurrently, there was a stakeholder engagement across the province in refreshing and expanding a least restraint guideline for children and youth in hospital inpatient and emergency and urgent care settings, and the selected quality measure would provide the opportunity to evaluate the roll out of an updated practice across the province.

The quality measure that was ranked the highest in our study analyzes perceived levels of satisfaction with support following discharge from an inpatient admission for mental health or substance misuse (47). This is consistent with the participants' comments on the importance of having patient and family voices (teenagers and parents in our case) represented in the final set of quality measures, in addition to their active participation in the Delphi selection process. Including patient-reported outcomes has increasingly been recognized as an important component of a quality measurement program and framing the pediatric approach to quality (20). Key to the successful involvement of patient and family partners was spending time to support their engagement. This was facilitated by having preparatory meetings in which the purpose, aim and methodology of the study were explained. Ensuring they had a point person who supported them during the discussion was also critical, in case the conversation proved to be triggering and they needed follow-up support after the session. The inclusion of patient and family participants provided key insights and value to the discussion round and the ranking and selection of the final five quality measures.

As a next step, the five top-ranked pediatric mental health quality measures will move into a measure refinement and testing phase, and processes for provincial data sharing, analysis, interpretation and reporting will be developed concurrently. With expected variation across the province, measures will be tested for technical feasibility, reliability, sensitivity to change, acceptability and implementation issues (54). This step is crucial because a developed theoretically sound indicator set may not work in practice (23). In our study, participants were asked about the expected feasibility of data collection for each measure. The results were not counted in the cut-off thresholds–to avoid that measures were chosen depending on the effort required to collect them–but will be used to inform the testing phase. As Evans et al. (23) point out, many indicator sets are based on the ease of data collection; fewer are based on sound epidemiological principles or a purpose-designed data system.

Different types of analyses will be conducted in the implementation phase. As Scobie et al. (55) suggest, a variety of risk adjustments (including sociodemographic and clinical characteristics) are needed to make the raw data more specific and meaningful. They also state that measures can support the understanding of local variations and act as a screen to determine or identify areas for further local analysis, potentially generating new hypotheses (55). Some of the selected measures, such as the proportion of children or young people who visited the ED for a mental health or substance-related disorder (50), will also be analyzed by assigned Tiers of Service. This Tiers of Service framework assists in the planning and coordination of mental health services provincially, within and across health authorities and multiple sectors and service providers (18). A high level of collaboration across networks of primary, secondary and social care services is key to delivering appropriate and continuous care to patients with mental illnesses (56). Utilizing tiers to analyze the data across the province will support understanding of similarities and variations of same-tiered mental health services. The implementation of the quality measures will therefore not only help to better understand local differences but also provide a provincial perspective on where collaborations are needed to support a better patient experience and continuity of care.

The five mental health quality measures will form part of an envisioned broader provincial core set of pediatric quality measures and will include measures related to child and youth populations living with asthma, diabetes and medical complexity. These measures will be added using a similar process over the next few years. The core set of pediatric quality measures will act as a foundation upon which to support greater transparency across the system and engage in mutually beneficial shared learning conversations to improve the quality of care. Consistent with a Learning Health System approach, having available data is key to understanding potential problems and identifying opportunities leading to innovative design, implementation and evaluation to influence and drive continuous improvement (57).

Three study limitations warrant consideration. First, in the Delphi study, the response rate was 50% or less, depending on the round. This is probably due to two main reasons: (i) one of the two expert groups that was contacted also participated in another provincial mental health project and therefore may have viewed participation as a competing priority to their available time for other provincial work; and (ii) the study took place during the COVID-19 pandemic where participants struggled to keep up with the increased need for mental health services and had less time available for quality improvement projects. We decided not to recruit additional participants as the participants from the two pre-existing stakeholder groups had diverse representation of both roles and geographical distribution, and we observed that participants were particularly engaged given the relevance of the work and being directly affected by the decisions made (35). Adjustments made to the methodology to comply with Covid-19 pandemic measures, such as organizing virtual conferences to discuss the results between the rating rounds, typically done in face-to-face meetings (32), and the use of Mentimeter, a real-time voting tool, facilitated the process and ensured study success.

The second study limitation also relates to stakeholder recruitment and participation, particularly within the pre-selection phase. In recruiting an expert group, there was a tension between keeping the group small enough to ensure each individual's participation within the discussion round, thereby keeping the time commitment manageable, balanced with wanting all health regions and diverse roles respresented to provide unique insights. This tension resulted in limited opportunities for duplication of representative roles within the expert group. Concern was expressed by stakeholders that important roles such as psychiatrists from across the province were not adequately represented. This limitation was addressed in the Delphi rounds, where an expanded group of mental health stakeholders assessed the pre-selected quality measures against three relevance criteria.

The third study limitation refers to the reliance on quality measures, which were contained within existing indicator or quality measure databases and datasets. These measures had to be validated or were being used within large quality measurement programs (i.e., National Institute for Health and Care Excellence quality standards and indicators). It is acknowledged that to date only small investments have been made in the development of pediatric quality measures (20), predominantly related to the greater number and cost of adults accessing and utilizing health services. Within that number of pediatric measures, an even smaller number are specific to mental health and substance use. Developing new measures and completing the validation process was not feasible due to limited resources. Nonetheless, a sufficient number was identified to be able to select a suite of relevant measures to be implemented in the province.

Through the application of a modified Delphi technique, provincial stakeholders selected pediatric mental health measures that will be implemented across the province. The five top-ranked measures will be refined for consistency and tested for data collection feasibility and processes for provincial data sharing, analysis, interpretation and reporting are being developed concurrently. Building upon this foundation, additional measures will be identified over the next years as part of a broader provincial core set of pediatric quality measures and will include measures related to child and youth populations living with asthma, diabetes and medical complexity. The use of Delphi study results has led to important advances in a range of practices in the mental health field (25) and our study contributes to it by implementing the selected core set of quality measures that has the potential to ultimately improve mental health services for children and youth across the province of British Columbia.

## Data Availability Statement

The original contributions presented in the study are included in the article/Supplementary Material. Further inquiries can be directed to the corresponding author/s.

## Ethics Statement

The Alberta Research Ethics Community Consensus Initiative (ARECCI) (58) ethics screening tool as well as the Provincial Health Services Authority Research and Academic Services determined that a formal ethics review was not needed given that the project was judged to be quality improvement rather than research.

## Author Contributions

RJ was the principal investigator and together with SW, WW, MS, and LJ designed the study and collected and interpreted the data. SW conducted the analyses and wrote the first draft of the manuscript. WW, MS, LJ, and RJ substantially contributed to improving the manuscript. All authors read and approved the final version before submission.

## Conflict of Interest

The authors declare that the research was conducted in the absence of any commercial or financial relationships that could be construed as a potential conflict of interest.

## Publisher's Note

All claims expressed in this article are solely those of the authors and do not necessarily represent those of their affiliated organizations, or those of the publisher, the editors and the reviewers. Any product that may be evaluated in this article, or claim that may be made by its manufacturer, is not guaranteed or endorsed by the publisher.
