# Supplementary material for: Selection of Pediatric Mental Health Quality Measures for Health System Improvement in British Columbia Based on a Modified Delphi Approach
Source: Front Pediatr. 2022 Jul 6;10:866391. doi: 10.3389/fped.2022.866391 (PMC9298984; doi:10.3389/fped.2022.866391)
Supplement: Supplementary file 1 [file Table_1.pdf]

Supplementary Material

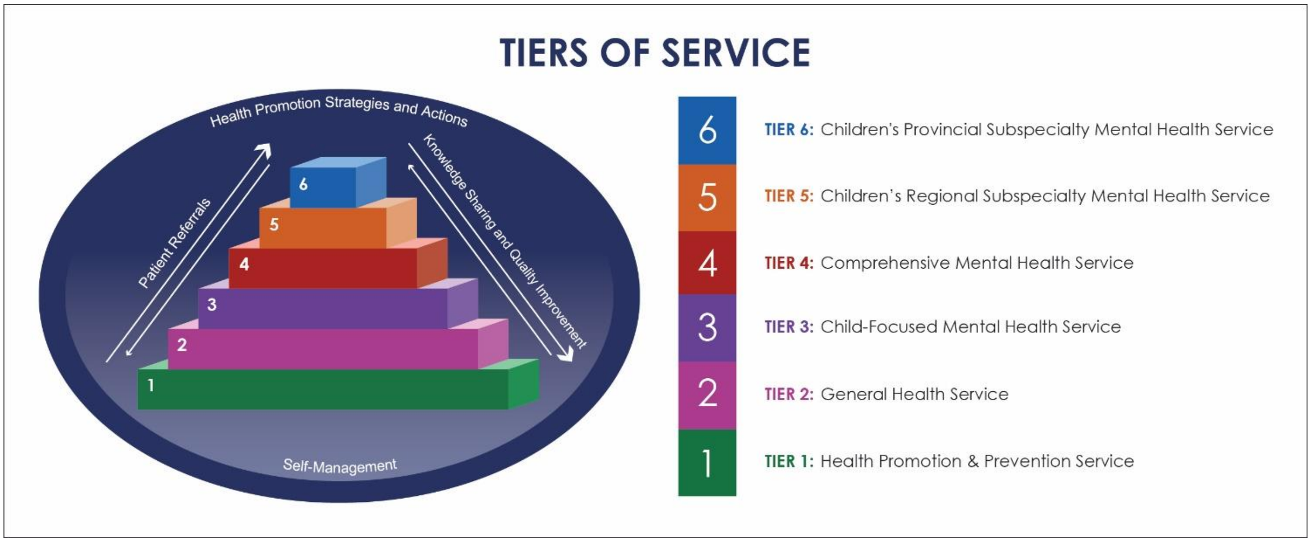

Supplementary Figure 1. Tiers of Service framework for mental health services for children and youth

Supplementary Table 1. Key features of Tiers 2-6 for children's mental health services

Area	Tier 2<br>General health services	Tier 3<br>Child-focused health services	Tier 4<br>Children's comprehensive health services	Tier 5<br>Children's regional enhanced subspecialty health services	Tier 6<br>Children's provincial subspecialty health services
**Hospital inpatient**	Short-term inpatient stays for children/youth up to age 18.9 yrs. Accommodated in a non pediatric-specific bed on a general inpatient unit. Service focus is on stabilization & crisis intervention. Anticipated length of stay is <72 hrs. By 72 hrs, child/youth will be discharged home with appropriate community MH services or transferred to higher tier. Applicable to rural/remote hospitals	Inpatient stays for children/youth up to age 16.9 yrs. Accommodated in a pediatric-specific bed on a general inpatient unit. Service focus is on stabilization & crisis intervention. Anticipated length of stay is <72 hrs. By 72 hrs, child/youth will be discharged home with appropriate community MH services or transferred to higher tier.<br>Clearly describable process is in place for managing youth ages 17 - 18.9 yrs with MH conditions on a general inpatient or alternative unit.	Where no T5 specialized child & adolescent psychiatry unit exists locally (i.e., within the same community), inpatient stays for children/youth up to age 16.9 yrs. Accommodated on a pediatric-specific inpatient unit. Service focus is on stabilization & crisis intervention. Anticipated length of stay is <72 hrs. By 72 hrs, child/youth will be discharged home with appropriate community MH services or transferred to higher tier.<br>Where T5 child & adolescent psychiatry unit exists locally, admission is arranged to this specialized unit.<br>Clearly describable process is in place for managing youth ages 17 - 18.9 yrs with MH conditions on a general inpatient or alternative unit.	Inpatient stays for youth up to age 18.9 yrs. Accommodated on a specialized child & adolescent psychiatry unit. Service focus is:<br>• Children up to 11.9 yrs: Stabilization & crisis intervention. Length of stay may be >72 hrs.<br>• Children 12 - 18.9 yrs: Stabilization & crisis intervention & ongoing treatment.	Inpatient stays for children/youth up to age 18.9 yrs. Accommodated on one of several subspecialty child & adolescent psychiatry inpatient units.<br>Service focus includes stabilization & crisis intervention & ongoing treatment for children/youth of all ages.
**Community-based & ambulatory**		Community-based providers provide psychoeducation, skill	Community-based interdisciplinary Child & Youth MH (CYMH)	Community or hospital outpatient-based, interdisciplinary teams of	Hospital outpatient-based, interdisciplinary, subspecialty MH teams

Area	Tier 2<br>General health services	Tier 3<br>Child-focused health services	Tier 4<br>Children's comprehensive health services	Tier 5<br>Children's regional enhanced subspecialty health services	Tier 6<br>Children's provincial subspecialty health services
		building & coaching to support recovery/ coping. Support access to follow-up care for MH &/or medical condition(s). Services may be provided in a range of settings such as child/youth's home, school or an office in the community.	Teams assess, diagnose & treat children/youth with a broad range of moderate acuity/complexity MH conditions/concurrent disorders. Teams provide case management & service coordination for children/youth involved with the service.<br>Where sufficient volumes exist (i.e., urban settings), dedicated teams provide short-term, assessment & crises intervention outreach services (e.g., in home or in community settings).<br>Where volumes are insufficient, a clearly describable process exists for providing these services in alternative ways (e.g., virtually or in local ED).	subspecialty MH providers assess, diagnose & treat children/youth with relatively common high acuity &/or high complexity MH conditions/concurrent disorders. Most children/youth will return to T4 for ongoing follow-up. Teams/clinics include but are not limited to:<br>• Infant psychiatry (5 yrs old & younger)<br>• Eating disorders<br>• Externalizing behavioral disorders<br>• Mood/anxiety<br>• Neurodevelopmental disorders with comorbid MH condition(s). Where sufficient volumes exist (i.e., urban settings), home-based & day treatment services are available.	assess, diagnose & treat children/youth with a broad range of high acuity &/or high complexity MH conditions/concurrent disorders. Focus is on children & youth with severe, complex &/or persistent MH conditions which have not responded with T2-T5 services. Medical co-morbidities often present & require monitoring/ treatment by one or more medical/surgical pediatric subspecialists. Most children/youth will return to T4 for ongoing follow-up.
**Residential**			Residential placement in a foster family, kinship or group home for children and youth in Ministry of Children & Family Development (MCFD) care. Placements are not specific to children/youth with MH conditions +/- behavioral concerns.<br>MH assessment &	Residential assessment & treatment service provided in a specialized, staffed group home. i.e., MCFD-contracted Complex Care Community Residential Resource.<br>Service focuses on behavior stabilization & on teaching children/youth/families	Residential assessment & treatment service provided in a community-based, facility setting. Includes a unit which provides step-up (avoid hospitalization) and step-down (transition out of hospital care) care.<br>Service focuses on children & youth with complex & often co-occurring &

**Area**	**Tier 2** General health services	**Tier 3** Child-focused health services	**Tier 4** Children's comprehensive health services	**Tier 5** Children's regional enhanced subspecialty health services	**Tier 6** Children's provincial subspecialty health services
			treatment services required while in T4 residential placement are provided through community-based & ambulatory services.	about techniques for managing challenging behaviors at home.	recurring conditions.

Supplementary Table 2. Identified indicators in database search, focus area inclusion, and expert panel pre-selection

Focus area	Source	Mental health quality measure	Included in selected focus area (Phase 2)	Pre-selected by expert group (Phase 3)
**Access / Sub-specialty Access**	Agency for Health Care Research and Quality (AHRQ) and National Quality Measures Clearinghouse (NQMC)	1. Access to outpatient child and adolescent psychiatrists, neurodevelopmental pediatricians, and developmental-behavioral pediatricians	Yes (included in measure #13; limited to access to psychiatrist)	No
	Canadian Institute for Health Information (CIHI)	2. Number of children who visited the emergency department for mental disorders	Yes (included in measure #4)	No
		3. Number of children and youth hospitalized for mental disorders	Yes (included in measure #5)	No
		4. Children and youth who visited the emergency department for mental disorders, by diagnosis and age group, selected jurisdictions, and rural/urban residence	Yes	Yes
		5. Children and youth hospitalized for mental disorders, by diagnosis and age group	Yes	Yes
	Expert panel addition	6. Number of emergency department visits of children/young people whose presenting complaint is behaviour	-	Yes
		7. Number of children/young people brought to the emergency department by police under Section 28 of the Mental Health Act where the presenting complaint is behaviour due to a mental health disorder	-	Yes
		8. Number of children/young people admitted who have pre-existing community supports	-	Yes
	Ontario MH of CY 2017	9. Number of outpatient physician visits related to mental health and addictions per 100 crude population aged 0 to 24 years, by age group and physician specialty, in Ontario, three-year average for 2012 to 2014	Yes	No
		10. Number of emergency department visits related to mental health and addictions per 1,000 crude population aged 0 to 24 years, by age group, 2006 to 2014	Yes (included in measure #4)	No
		11. Number of hospitalizations related to mental health and addictions per 1,000 crude population aged 0 to 24 years, by age group, in Ontario, 2006 to 2014	Yes (included in measure #5)	No
		12. Number of children and youth for whom the ED was the first point of contact for MHA care per 100 crude population aged 0 to 24 years with an MHA-related ED visit, by type of disorder, in Ontario, three-year average for 2012 to 2014	Yes	No
		13. Number of children and youth seen by a psychiatrist per 1,000 population	Yes	No

Focus area	Source	Mental health quality measure	Included in selected focus area (Phase 2)	Pre-selected by expert group (Phase 3)
		aged 0 to 24 years, overall and by sex, in Ontario, 2006 to 2014		
		14. Number of children and youth for whom the ED was the first point of contact for MHA care per 100 population aged 0 to 24 years with an MHA-related ED visit, overall and by sex, in Ontario, 2006 to 2014	Yes (included with measure #12)	No
		15. Number of ED visits and hospitalizations related to mental health and addictions per 1,000 crude population aged 0 to 24 years, by neighborhood income quintile, in Ontario, three-year average for 2012 to 2014	Yes (included in measure #4 & #5)	No
		16. Number of ED visits and hospitalizations related to mental health and addictions per 1,000 crude population aged 0 to 24 years, by immigrant category, in Ontario, three-year average for 2010 to 2012	No (BC did not collect race data at time of study)	No
		17. Number of children and youth seen by a psychiatrist per 1,000 standard population aged 0 to 24 years, by Local Health Integration Network, in Ontario, three-year average for 2012 to 2014	No (not applicable for BC)	No
**ADHD**	Danish Government - Clinical quality development program	18. Proportion of patients of 6-18 years, undergoing a manualized diagnostic interview concerning differential diagnosis and comorbidity either Kiddie-Sad, PSE-SCAN or DAWBA within 90 days of ADHD investigation	No	No
	National Committee for Quality Assurance (NCQA); Healthcare Effectiveness Data and Information Set (HEDIS)	19. Percentage of members 6 to 12 years of age with an ambulatory prescription dispensed for ADHD medication who remained on the medication for at least 210 days and who, in addition to the visit in the initiation phase, had at least two follow-up visits with a practitioner within 270 days (9 months) after the initiation phase ended *	No	No
		20. Percentage of members 6 to 12 years of age with an ambulatory prescription dispensed for ADHD medication who had one follow-up visit with a practitioner with prescribing authority during the 30-day initiation phase *	No	No
**Admission screening**	Agency for Health Care Research and Quality (AHRQ) and National Quality Measures Clearinghouse (NQMC)	21. Percentage of patients admitted to a hospital-based inpatient psychiatric setting who are screened within the first three days of admission for all of the following: risk of violence to self or others, substance use, psychological trauma history and patient strengths *	No	No
**Anti-psychotic medication use**	Agency for Health Care Research and Quality (AHRQ) and National Quality Measures Clearinghouse (NQMC)	22. Children/adolescents admitted to the hospital with a diagnosis of psychotic disorder that have documentation in their medical record of a baseline metabolic assessment prior to starting any scheduled antipsychotic medication that includes: height, weight, blood pressure, pulse, blood glucose, total cholesterol, triglycerides and EKG	No	No
		23. Children/adolescents admitted to the hospital with psychotic symptoms	No	No

Focus area	Source	Mental health quality measure	Included in selected focus area (Phase 2)	Pre-selected by expert group (Phase 3)
		who are not currently taking antipsychotics that have a psychiatric consult prior to initiating scheduled treatment with antipsychotic medications		
		24. Use of higher than recommended doses of antipsychotics in children and adolescents	No	No
		25. Percentage of patients discharged from a hospital-based inpatient psychiatric setting on two or more antipsychotic medications with appropriate justification *	No	No
	Canadian Institute for Health Information (CIHI)	26. Youth dispensed psychotropic medications, by medication category (Mood = SSRI, Tricyclic antidepressant, Other antidepressant, MAOI, Anxiolytic, Lithium) (Antipsychotic: Typical, Atypical) and selected jurisdiction	No	No
	Medicaid Child Core Set 2016 and 2020	27. Percentage of use of two or more concurrent antipsychotic medications in children ages 1 to 17	No	No
	National Quality Forum (NQF)	28. Percentage of children under age 5 who were dispensed antipsychotic medications during the measurement period	No	No
		29. Percentage of children and adolescents 1–17 years of age who had two or more antipsychotic prescriptions and had metabolic testing	No	No
		30. Percentage of children and adolescents 1–17 years of age with a new prescription for an antipsychotic, but no indication for antipsychotics, who had documentation of psychosocial care as first-line treatment	No	No
**Anxiety disorders**	National Institute for Health and Care Excellence (NICE)	31. Proportion of people with a suspected anxiety disorder who receive an assessment that identifies whether they have a specific anxiety disorder, the severity of symptoms and associated functional impairment	No	No
**Disruptive behavior disorders, conduct disorder, oppositional defiant disorder**	National Institute for Health and Care Excellence (NICE)	32. Proportion of children and young people with a suspected conduct disorder and any significant complicating factors who have a comprehensive assessment	Yes	No
		33. Proportion of children and young people with a conduct disorder and severely aggressive behavior who have been prescribed risperidone who have a baseline physical and metabolic investigation carried out and recorded before the start of treatment	Yes	No
**Eating disorders**	National Institute for Health and Care Excellence (NICE)	34. Length of time from referral to assessment and start of treatment at an eating disorder service for children and young people with suspected eating disorders	Yes	Yes
		35. Proportion of people with eating disorders who are supported by more than one service who have a care plan that explains how the services will work together	Yes	No

Focus area	Source	Mental health quality measure	Included in selected focus area (Phase 2)	Pre-selected by expert group (Phase 3)
		36. Rate of relapse for people with eating disorders who are supported by more than one service	Yes	No
		37. Proportion of children and young people with suspected eating disorders who start assessment and treatment within a maximum of 4 weeks from first contact with a designated healthcare professional	Yes (used measure #34 instead)	No
		38. Rate of recovery for people with eating disorders	No (no clear definition of recovery)	No
		39. Proportion of people with eating disorders who have a documented discussion with a healthcare professional about their options for psychological treatment at diagnosis	No	No
		40. Proportion of people with eating disorders who are supported by more than one service and have CPA meetings to discuss their care plan	No (not applicable to BC context)	No
		41. Service user experience of eating disorder services	No	No
		42. Proportion of people with eating disorders moving between services who have a care plan that includes a risk assessment before transfer	Yes (used measure #35 as general measure)	No
		43. Proportion of people with eating disorders moving between services who have a care plan that includes a risk assessment after transfer	Yes (used measure # 35 as general measure)	No
		44. Proportion of people with eating disorders who have moved between services and did not attend their first meeting or appointment	No	No
		45. Relapse rate of people with eating disorders who move between services	Yes (used measure #36 as general measure)	No
	Expert panel addition	46. Eating Disorder severity: Body Mass Index at time of admission	-	No
	Ontario MH of CY 2017	47. Number of hospitalizations for eating disorders per 10,000 population aged 7 to 24 years, overall and by sex, in Ontario, 2003 to 2014 (up to age 18.9), and change to gender	Yes	Yes
**Mental Health Act compliance**	Government of British Columbia	48. Percentage of pediatric mental health inpatients who have their form 4 completed on the day of admission	No	No
		49. Percentage of pediatric mental health inpatients who have their form 14 or 13 completed within 24h of completion of the 1st Form 4 and admission to the unit.	No	No
		50. Proportion of youth who were informed were informed of their rights under the Mental Health Act promptly upon admission	No	No

Focus area	Source	Mental health quality measure	Included in selected focus area (Phase 2)	Pre-selected by expert group (Phase 3)
**Mood disorders (depression/bipolar)**	Agency for Health Care Research and Quality (AHRQ) and National Quality Measures Clearinghouse (NQMC)	51. Utilization of the PHQ-9 to monitor depression symptoms for adolescents and adults	No	No
	National Institute for Health and Care Excellence (NICE)	52. Proportion of children and young people with suspected depression who have a diagnosis confirmed and recorded in their medical records	No	No
		53. Proportion of children and young people with depression who are given information appropriate to their age about the diagnosis and their treatment options	No	No
		54. Evidence from experience surveys and feedback that children and young people with depression understand the diagnosis and their treatment options	No	No
		55. Evidence of local arrangements to ensure that CAMHS professionals assess all children and young people with suspected severe depression and at high risk of suicide within a maximum of 24 hours of referral	No	No
		56. Proportion of children and young people with suspected severe depression and at high risk of suicide who are assessed by CAMHS professionals within 24 hours of referral	No	No
		57. Proportion of children and young people with suspected severe depression but not at high risk of suicide who are assessed by CAMHS professionals within 2 weeks of referral	No	No
	National Quality Forum (NQF)	58. Percentage of patients aged 12 years and older screened for depression on the date of the encounter or 14 days prior to the date of the encounter using an age appropriate standardized depression screening tool AND if positive, a follow-up plan is documented on the date of the eligible encounter	No	No
		59. Percentage of patients aged 6 through 17 years with a diagnosis of major depressive disorder with documented evidence that they met the DSM-IV criteria [at least 5 elements with symptom duration of two weeks or longer, including 1) depressed mood or 2) loss of interest or pleasure] during the visit in which the new diagnosis or recurrent episode was identified	No	No
		60. Percentage of patient visits for those patients aged 6 through 17 years with a diagnosis of major depressive disorder with an assessment for suicide risk	No	No
		61. Percentage of adolescents 13 years of age who had a screening for	No	No

Focus area	Source	Mental health quality measure	Included in selected focus area (Phase 2)	Pre-selected by expert group (Phase 3)
		depression using a standardized tool		
		62. Percentage of adolescents 18 years of age who had a screening for depression using a standardized tool	No	No
**Neuro-diverse conditions**	National Institute for Health and Care Excellence (NICE)	63. Proportion of people with neurodiverse conditions who develop behavior that challenges who are assessed for possible triggers, including physical health conditions, mental health problems and environmental factors	Yes	Yes
		64. Evidence of local arrangements to monitor the use of antipsychotic medication in people with neurodiverse conditions that challenges	Yes	No
		65. The proportion of people with autism and behavior that challenges having antipsychotic medication for the treatment of their behavior that challenges in whom psychosocial interventions are insufficient or cannot be delivered because of the severity of the behavior	Yes	Yes
**Personality disorders**	Canadian Institute for Health Information (CIHI)	66. Number of Children and youth who visited the emergency department with a diagnosis of personality Disorder by age, jurisdiction, rural/urban residence	No	No
**Provider education (including training, clinical supervision & support)**	Expert panel addition	67. The percentage of staff that have completed training in least restraints guidelines as per Health Authority (initial and annual training)	-	Yes
	Government of British Columbia: Mental Health Act Compliance	68. Percentage of staff that have Mental Health Act face-to-face session completed (synchronous; may be delivered virtually)	Yes (combined with measure #69)	Yes (combined with measure #66 & 68)
		69. Percentage of new staff that have an online Mental Health Act module completed within one month of hire	Yes (combined with measure #68)	No
		70. Percentage of staff that have culture safety training completed (San'yas or similar)	Yes	No
	Medicaid Child Core Set 2016 and 2023	71. Percentage of staff that have Mental Health Act online module completed (asynchronous)	Yes (included with measure #68)	No
	National Institute for Health and Care Excellence (NICE)	72. Evidence of structures to ensure that healthcare professionals receive training to perform assessments of e.g. anxiety disorders, mood disorders	Yes	No
**Psychosocial/ family functioning**	Agency for Health Care Research and Quality (AHRQ) and National Quality Measures Clearinghouse (NQMC)	73. Proportion of children whose parents were assessed for one or more topics related to psychosocial well-being *	Yes	Yes
**Psychotic & schizo-phrenic**	Agency for Health Care Research and Quality (AHRQ) and National	74. Children/adolescents admitted to the hospital with psychotic symptoms that have a psychiatric consult (in person or tele-psychiatry) within 24 hours of admission	No	No

Focus area	Source	Mental health quality measure	Included in selected focus area (Phase 2)	Pre-selected by expert group (Phase 3)
**disorders**	Quality Measures Clearinghouse (NQMC)			
	National Quality Forum (NQF)	75. Percentage of children/adolescents age 5 to 19 years old seen in the emergency department with psychotic symptoms who are screened for alcohol or drugs of abuse	No	No
**Restraint/ seclusion**	Agency for Health Care Research and Quality (AHRQ) and National Quality Measures Clearinghouse (NQMC)	76. Total number of hours that all patients admitted to a hospital-based inpatient psychiatric setting were maintained in physical restraint *	Yes	No
		77. Total number of hours that all patients admitted to a hospital-based inpatient psychiatric setting were held in seclusion *	Yes	No
	Expert panel addition	78. The total number of children/young people who experienced at least one event of seclusion or restraint (physical and chemical) during their stay.<br>a) The number of children/young people who experienced at least one seclusion, restraint or both (reported separately)<br>b) The number of seclusions, restraint or both events per patient stay (reported separately)	-	Yes
		79. Number of hours in seclusion/restraint over total hours of hospital inpatient stay	-	Yes
**Self-harm**	Agency for Health Care Research and Quality (AHRQ) and National Quality Measures Clearinghouse (NQMC)	80. Children/adolescents who present to the ED with dangerous self-harm or suicidality who are discharged to home should see a mental health professional for follow-up within 7 days of discharge from the ED	Yes	No
		81. Children/adolescents admitted to the hospital for dangerous self-harm or suicidality that have documentation in the hospital record that showed communication and planning between acute and outpatient providers for discharge follow-up.	Yes	Yes
		82. Children/adolescents who present to the ED with dangerous self-harm or suicidality who are discharged to home and have an assessment by a mental health professional prior to discharge from the ED	Yes	No
		83. Children/adolescents who were admitted to the hospital for dangerous self-harm or suicidality that have documentation in the hospital record that their caregiver was counseled on how to restrict the child's/adolescent's access to potentially lethal means of suicide (e.g., firearms, medications, car, etc.) prior to discharge	Yes	No
	Expert panel addition	84. Number of readmissions to ED with a presenting complaint of self-harm within 7/30 days	-	Yes (combined with measure #105)
**Substance**	Canadian Institute for	85. Proportion of children and youth who visited the emergency department	Yes	Yes

Focus area	Source	Mental health quality measure	Included in selected focus area (Phase 2)	Pre-selected by expert group (Phase 3)
**use/substance -related disorders**	Health Information (CIHI)	for a substance-related disorder by age, jurisdiction, rural/urban residence		
	National Quality Forum (NQF)	86. Percentage of emergency department visits for members 13 years of age and older with a principal diagnosis of alcohol or other drug (AOD) abuse or dependence, who had a follow up visit for AOD. Two rates are reported:<br>- Percentage of ED visits for which the member received follow-up within 30 days of the ED visit (31 total days)<br>- Percentage of ED visits for which the member received follow-up within 7 days of the ED visit (8 total days)	Yes	Yes
		87. Percentage of patient aged 12 to 18.9 years old who were admitted with mental health or substance misuse diagnosis that received a referral to addiction treatment services or/and medication for addiction treatment upon discharge	Yes	No
		88. Percentage of patients aged 12 to 18.9 years old who were screened for substance use at least once during a hospital admission with mental health or substance use diagnosis	Yes	No
**Suicide**	Government of British Columbia	89. Number of deaths by suicide per 100,000 standard population aged 10 to 24 years, by Health Service Delivery Area	No	No
	Ontario MH of CY 2017	90. Number of deaths by suicide per 100,000 standard population aged 10 to 24 years, by Local Health Integration Network, in Ontario, three-year average for 2007 to 2009 and 2010 to 2012	No	No
**Transition / discharge / Follow-up**	Agency for Health Care Research and Quality (AHRQ) and National Quality Measures Clearinghouse (NQMC)	91. Timeliness of follow up visits following hospital discharge of children with a primary mental health diagnosis	Yes	No
	Medicaid Child Core Set 2016 and 2021	92. Percentage of discharges with a follow-up visit within 30 days, after hospitalization for mental illness, 6-20 years	Yes (included in measure #102)	No
	Medicaid Child Core Set 2016 and 2021	93. Percentage of discharges with a follow-up visit within 7 days, after hospitalization for mental illness, 6-20 years	Yes (included in measure #102)	No
	National Institute for Health and Care Excellence (NICE)	94. Proportion of admissions to an inpatient mental health setting for which information is provided on admission about support available from independent advocacy services	Yes	No
	National Institute for Health and Care Excellence (NICE)	95. Level of satisfaction with access to independent advocacy services for people using inpatient mental health settings	Yes	No
		96. Evidence of local arrangements to send within 24 hours, the care plans of	Yes	No

Focus area	Source	Mental health quality measure	Included in selected focus area (Phase 2)	Pre-selected by expert group (Phase 3)
	National Quality Forum (NQF)	people discharged from an inpatient mental health setting to everyone identified in it as involved in their ongoing care		
		97. Proportion of discharges from an inpatient mental health setting where the person's care plan is sent within 24 hours to everyone identified in it as involved in their ongoing care	Yes	No
		98. Level of satisfaction with support following discharge from inpatient mental health settings	Yes	Yes
		99. Readmissions to inpatient mental health services within 30 days of discharge	Yes	Yes
		100. Proportion of discharges from an inpatient mental health setting in which people are followed up within 48 hours of discharge if they are identified as being at risk of suicide	Yes	No
		101. Number of people who have either died by suicide or experienced a suicide attempt who were discharged within 3 months of an inpatient mental health admission	Yes	No
		102. Percentage of discharges for patients 6 years of age and older who were hospitalized for treatment of selected mental illness diagnoses and who had a follow-up visit with a mental health practitioner. Two rates are reported:<br>- Percentage of discharges for which the patient received follow-up within 30 days of discharge<br>- Percentage of discharges for which the patient received follow-up within 7 days of discharge.	Yes	No
	Ontario MH of CY 2017	103. Number of outpatient visits within 7 days of an incident hospital discharge related to MHA per 100 population aged 0 to 24 years with an incident MHA-related hospital discharge, by physician specialty and sex, in Ontario, 2006 to 2014	Yes (included in measure #102)	No
		104. Number of outpatient visits within 7 days of an incident hospital discharge related to MHA per 100 population aged 0 to 24 years with an incident MHA-related hospital discharge, by hospital type and weighted by hospital discharge volume, in Ontario, 2014	Yes (included in measure #102)	No
		105. Number of ED revisits within 30 days of an incident ED visit related to MHA care per 100 population aged 0 to 24 years with an incident MHA-related ED visit, overall and by sex, in Ontario, 2006 to 2014	-	Yes (combined with measure #8)

Focus area	Source	Mental health quality measure	Included in selected focus area (Phase 2)	Pre-selected by expert group (Phase 3)
		106. Number of readmissions within 30 days of an incident hospital admission related to MHA per 100 population aged 0 to 24 years with an incident MHA-related hospital admission, overall and by sex, in Ontario, 2006 to 2014	Yes (included in measure #99)	No
		107. Number of ED revisits within 30 days of an incident ED visit related to MHA per 100 population aged 0 to 24 years with an incident MHA-related ED visit, by hospital type and weighted by ED discharge volume, 2014	No (included in measure #105)	No
		108. Number of readmissions within 30 days of an incident hospital admission related to MHA per 100 population aged 0 to 24 years with an incident MHA-related hospital admission, by hospital type and weighted by hospital discharge volume, 2014	Yes (included in measure #99)	No

* Endorsed by the National Quality Forum (NQF)
BC; British Columbia
